# Double Jeopardy-Analyzing the Combined Effect of Age and Gender Stereotype Threat on Older Workers

**DOI:** 10.3389/fpsyg.2020.606690

**Published:** 2021-01-12

**Authors:** Claudia Manzi, Angela Sorgente, Eleonora Reverberi, Semira Tagliabue, Mara Gorli

**Affiliations:** ^1^Università Cattolica del Sacro Cuore, Milan, Italy; ^2^Università Cattolica del Sacro Cuore, Brescia, Italy

**Keywords:** gender stereotype threat, age-based stereotype threat, work identity, workplace, organization, HR management

## Abstract

In this study we aim to analyze the combined effect of age-based and gender stereotype threat on work identity processes (and in particular on authenticity and organizational identification) and on work performance (self-rating performance). The research utilizes an ample sample of over fifty-year-old workers from diverse organizations in Italy. Using a person-centered approach four clusters of workers were identified: low in both age-based and gender stereotype threat (*N* = 4,689), high in gender and low in age-based stereotype threat (*N* = 1,735), high in age-based and low in gender stereotype threat (*N* = 2,013) and high in both gender and age-based stereotype threat (*N* = 758). Gender was significantly associated with these clusters and women were more frequently present in those groups with high gender stereotype threat. ANOVA results show that workers in the last two clusters score significantly lower in authenticity, organizational identification and self-rate performance. All in all, if ageism is undoubtedly problematic for older workers’ identity processes, ageism and gender-stereotypes represent a double risk for women over fifty in the workplace. The analysis of the results can be beneficial both for the theoretical advancement and for the practical insights offered in the organizational and management field, where new policies of HR management can be elaborated, in order to value and to improve the workers experience.

## Introduction

Stereotypes are beliefs about the characteristics, attributes, and behaviors of members of certain groups that simplify cognitive processes and help people cope with the complexity of the world (Hilton and von Hippel, 1996). However, these oversimplifications often affect how specific social groups are perceived, especially in the workplace. Discriminations that directly derive from negative stereotypes are particularly evident in areas such as employability, career opportunities, and income disparity (Deaux and Lafrance, 1998; Carli and Eagly, 1999; Perry and Finkelstein, 1999). In the workplace, discrimination is not the only negative effect of stereotype; a wealth of literature on the subject has shown that negative stereotypes are also the basis of an equally insidious phenomenon, the so-called “stereotype threat.”

Stereotype threat is defined as the belief that one may be the target of demeaning stereotypes (von Hippel et al., 2011a). In other words, stereotype threat describes the psychological pressure of a person who, while engaged in a task, is aware of one or multiple stereotypes about his or her identity group, suggesting that he or she will not perform well on that task (Roberson and Kulik, 2007). Empirical studies have documented the impact of stereotype threat. Evidence shows that when facing stereotype threat during a task, the ventral anterior cingulate cortex—a brain area associated with the control of emotions and the process of social feedback—is activated, leading to a drop in performance (Wraga et al., 2007; Krendl et al., 2008). The processes through which stereotype threat lead to a decrease in performance thus arise from increased anxiety, disturbing thoughts, overly cautious tendencies, pessimism, and disengagement (Maass and Cadinu, 2003). Anyone may experience a degree of anxiety while performing a task, but stereotype threat is known to place an additional load on members of the stereotyped groups. Victims of stereotype threat are said to feel “in the spotlight,” as their failure is not viewed as the result of a single performance but part of a foregone negative evaluation related to the larger group to which they belong.

This study focuses on age‐ and gender-based stereotype threat in the workplace and on its negative effect on older workers’ identity processes and performance. Previous studies have shown that both older workers and women are the target of negative stereotypes in the workplace (e.g., Sterns and Miklos, 1995; Taylor and Walker, 1998; Carli and Eagly, 1999; Eagly and Karau, 2002). However, to date, studies that analyze gender stereotype threat in conjunction with that of age are almost inexistent. Cleveland et al. (2017) have recently claimed that a combined consideration of age and gender may lead to a more accurate understanding of its effects on the work experience of employees over the age of 50. Therefore, the objective of this study is to expose these previously overlooked issues, analyzing both age and gender stereotype threat and their association with two key processes that directly affect the work experience: work identity processes and performance. The sample of this study included a wide range of workers over 50 years of age from various organizations operating in Italy. Using a person-centered approach, clusters of workers were identified according to age and gender stereotype threat. Moreover, differences in the rate of performance, authenticity, and organizational identification were tested across all the identified clusters. Through this research, we aim not only to advance the available theoretical knowledge on age and gender stereotype threat but also to offer practical insights for developing new organizational policies and practices to curb this occurrence.

### The Combined Effect of Gender and Age-Based Stereotype Threat in the Workplace

Data collected by the World Health Organization (2015) reveal that workers over 50—both men and women—are often subject to negative stereotypes associated with aging. Numerous pieces of research into age-based stereotypes in the workplace show that older workers are considered inflexible, unwilling to adapt to technology, resistant to change, physically limited, and more expensive for the organization (McCann and Giles, 2002). The stereotype threat that derives from this negative view of older workers has been found to produce adverse outcomes. Lamont et al. (2015) have documented that stereotype threat influences elderly workers especially on cognitive and memory tasks, rather than physical ones. This demonstrates that stereotype threat is particularly harmful within jobs that require intellectual efforts—typically office based—making the assessment of training needs for older workers quite challenging. Other studies have highlighted that stereotype threat disrupts task performance and negatively affects individuals’ motivation and engagement (Roberson and Kulik, 2007; Shapiro and Neuberg, 2007).

Yet, organizations are all-too-often scenarios of gender stereotypes. In an assessment that spanned over 40 years of research on gender stereotyping in the United States, United Kingdom, Germany, China, and Japan, Schein (2001) concludes that the notion “think leader–think male” is a universal phenomenon that has remained virtually unaltered since 1973, despite the enormous change in the composition of the global workforce. More recently, by comparing data collected in the early 1980s to new data collected in 2014, Haines et al. (2016) highlight that people perceive the same strong differences between men and women in stereotype components today as they did in the past. These results attest how powerful basic stereotypes still are, despite the significant shift in the participation and acceptance of women and men in nontraditional jobs. Thus, unsurprisingly, the stereotype threat on women is still an important impediment to gender equality and, in particular, to women’s perception of the workplace (for a review see Bobbitt-Zeher, 2011). For example, research shows that female employees that perceived a certain degree of stereotype threat have more negative job attitudes, recurrent desire to leave the company, and had reduced confidence that they could reach their career aspirations (von Hippel et al., 2011a).

The literature on the subject has often assumed implicitly that gender and age-based stereotype threats may operate independently. However, factors related to age combined with one’s gender may play a critical role in shaping her/his work life. Several research findings have underlined that ageism is perceived differently by women and men. Kornadt et al. (2013) showed that older women perceived age-related changes in most domains—family, friends, religion, leisure personality, and health—more positively than older men. However, women were less confident in the domains of finance and work, where evaluations traditionally favor older men. In line with these findings, researchers have found that women in the workplace experience more age-based stereotype threat than men (Manzi et al., 2019). This study demonstrates that age-based stereotype threat affects people differently according to gender. Nevertheless, what is still unavailable is an analysis of the combined effects of both gender and age-based stereotype threat experienced by people in their workplace. Following the intersectionality paradigm (Crenshaw, 1989), the study aims to focus on how this double jeopardy affects employees’ work experience by assessing two key aspects: identity processes and performance.

Methodologically, a person-centered approach was undertaken as part of the study. In this approach, the main areas of interest are the patterns that emerged among individuals rather than the linear relationships between variables (Bergman and Wångby, 2014). Thus, a person-centered approach allows us to answer the following questions—“What emergent subpopulations can be identified through the dimensions of age and gender stereotype threat?;” “How do these subpopulations differ in terms of outcomes?” As suggested by Meyer et al. (2013), one immediate practical benefit of using a person-centered approach is that managers have a natural inclination to think in terms of categories or types of people (vs. dimensions and variables). An analytic strategy that identifies subgroups of employees is potentially more compatible with the information processing approach of managers, who are often the ones in charge of enacting intervention personnel strategies. We believe that the approach used could help understand better the variables involved when considering workers and their characteristics. The cluster analysis will provide immediate reflections, also available to managers, aiming at influencing the introduction of policies that value, support, and improve the workers’ experience throughout the different phases of life, individual characteristics, or gender specificities. From a theoretical point of view, our study aims to contribute to the understanding of the intertwined effects of age and gender stereotype threats in organizational contexts.

### The Effects of Stereotype Threats on Workers’ Identity and Performance

A significant number of studies has shown how identity processes are a key aspect of human functioning. Identity is a never-ending process that is nurtured by multiple identifications and stages of life. Being part of an organization and feeling part of a group (or groups) is a key element of a mature identity process. The worker’s identity comprises of several dimensions. Two of these are the identification with the organization (Dutton et al., 1994) and the sense of authenticity. The latter measures how comfortable each worker feels about showing her/his true self in the workplace (van den Bosch and Taris, 2018). Typically, within the “Social Identity Theory” framework, the identification with social groups provides psychological well-being, as group identifications fulfill basic human needs—such as the need for belonging, self-esteem, and meaning—and provide a psychological ground on which to stand (see Haslam et al., 2009, for a review).

The identification with work organizations—i.e., “perception of oneness with or belongingness to an organization where the individual defines him or herself at least partly in terms of their organizational membership” (Mael and Ashforth, 1992, p. 109)—is thought to lead to positive psychological outcomes. In particular, the identification with one’s organization is linked to the fulfillment of several intrinsic needs—for example, the sense of safety, self-enhancement, and affiliation (Pratt, 1998). It is also associated with positive work outcomes such as commitment, performance, turnover, and retirement intentions (Van Knippenberg and van Schie, 2000; Abrams and Randsley de Moura, 2001; Riketta, 2005; Marique et al., 2013). Numerous studies have investigated the effects of stereotype threat on workers’ identification. Steele (1997) underlined that people who experience stereotype threat on a regular basis as an important life domain are likely to hide that identity domain through the process of “identity separation” (Steele et al., 2002). That means splitting the part of the identity that is perceived as being stereotyped in favor of another one. Identity separation in response to stereotype threat in the workplace has serious implications for workers’ health, as it increases the likelihood of developing depression, lowering the sense of self-fulfillment and self-esteem (Settles, 2004). It also generates disengagement and dis-identification toward the organization (von Hippel et al., 2011b). When experiencing negative stereotypes, employees direct their attention away from their job, decreasing their sense of identification with the organization and, in turn, their sense of citizenship (Wu et al., 2016). Consequently, we expect that cluster of workers experiencing higher levels of gender and age stereotype threat would report lower levels of organizational identification.

Together with organizational identification, we focused our attention on another dimension related to identity: authenticity. This construct has recently gained attention within the field of organizational studies. Empirical evidence shows that authenticity is a crucial aspect of workers’ life (e.g., van den Bosch and Taris, 2018). It is defined as the subjective experience of the alignment between one’s internal sense of self and their external expression (Caza et al., 2018). Authenticity fulfills the individuals’ need for self-esteem and promotes their sense of belonging to the company. Consequently, its direct link with well-being and work engagement is evident (Cha et al., 2019). Authenticity is strictly linked to the *fit* between people and their context, also defined as “a match between external characteristics of the environment and core characteristics of the individual” (see Schmader and Sedikides, 2018, p. 228). Higher levels of *fit* increase the probability that a person feels free to be authentic in a particular context. Conversely, experiencing negative stereotype in the workplace and lack of *fit* in one’s work context may be particularly threatening for workers’ authenticity. Members of social groups who are considered institutionally “less valued” may attempt to mask their stigmatized identity in the workplace (see for example Button, 2001; Ng et al., 2012). Therefore, it is reasonable to assume that workers who experience high levels of gender and age stereotype threat will display lower levels of authenticity.

Finally, we assessed the differences in performance rate within clusters of workers identified by levels of gender and age-based stereotype threat. A plethora of research has shown how stereotype threat leads to reduced performance in individuals of different target groups (i.e., Abrams et al., 2006; Nguyen and Ryan, 2008; Flore and Wicherts, 2015). In an experimental study carried out on retired workers, cognitive performance decreased as a direct effect of threats, and it deteriorated further in those individuals who had negative intergroup experiences with younger people (Abrams et al., 2006). Gender stereotype threat has also been found to impair women’s performance in different tasks (Flore and Wicherts, 2015); hence, it is fair to expect that clusters of workers that experience higher levels of stereotype threat display lower levels of performance.

### The Present Study

The study comes from the second wave of a research project called “*Talenti senza età*” (Ageless Talents) promoted by “Valore D,” an Italian association of companies that promotes diversity, talent, and female leadership to aid the economic growth of the country. “Ageless talents” is the first piece of research done on employed women and men over the age of 50 in Italy. It was conceived to provide some insights into this section of the working population and identify strategies aimed at supporting women over 50 in the workplace.

There is limited statistical data on ageism in the workplace in Italy. However, the little information available highlights several challenges. As emerged from a recent cross-cultural meta-analysis by North and Fiske (2015), Europe scored higher in ageism than the rest of the western countries and even higher than some parts of Asia. Moreover, this meta-analysis has also underlined a positive correlation between the aging rate and ageism. Italy is one of the world’s oldest countries, with the highest proportion of elderly people and the lowest proportion of young people, and this trend is set to continue in the future (OECD, 2017). Thus, by applying this trend to the findings of the study conducted by North and Fiske, Italy is likely to expect a growing level of ageism. In addition, the 2006 Kelly Global Workforce Index of over 70,000 workers in 28 countries (including Italy) provided some evidence of workforce age discrimination in Italy. More than half (58%) of the workers interviewed in Italy reported acts of age discrimination at the job application stage. This figure was among the highest in the 28 analyzed countries.

The condition of female employees in Italy is particularly alarming. The gender gap, especially in terms of career opportunity and salary, continues to widen, and the last 3 years we have seen a noticeable decline (World Economic Forum, 2017). In the global ranking on the gender gap, compiled in 2018, the World Economic Forum highlighted very low rates of gender pay equality in Italy, and as a result, Italy currently ranks 118th place out of 144 countries.

Together with this scenario, we must consider that the number of employed women aged between 55 and 64 in Italy has increased by 50% from 2010 to date (ISTAT, 2017). This increase is due to the pension reform—enacted by the “Fornero Law” in 2010—which extended retirement age for women from 60 to 65.

Women over the age of 50 in Italy are a growing section of the national labor force, and the difficult organizational context in which they operate exposes them to higher risk than other groups. Hence, it is extremely important to analyze the phenomenon of age and gender stereotype threat and its effect on well-being at work in order to safeguard the exposure of women to this debilitating occurrence.

By using a person-centered approach, the study aims to analyze clusters of Italian workers in relation to age and gender stereotype threat. Moreover, the differences in the rate of performance, authenticity, and organizational identification were tested across the identified clusters.

The exploratory nature of this study excludes *a priori* any hypotheses about the specific number of workers profiles that might emerge according to levels of gender and age stereotype threats. Nevertheless, based on the revised literature, we expect that the clusters of workers experiencing higher levels of gender and age-based stereotype threats would report lower levels of organizational identification, authenticity, and performance.

It is important to add that, in analyzing the data, the effect of gender and age was considered as a possible intervening variable in the composition of clusters of workers and the relationship between clusters and outcomes. Moreover, several studies (e.g., Hirsh and Kornrich, 2008; Gonzalez and Denisi, 2009) have also shown that when analyzing stereotype threat in the workplace, it is also important to focus on the characteristics of the company, i.e., size (number of employees), size of the minority group, etc. By taking into consideration these aspects, a possible effect of a company’s characteristics in the composition of clusters and the relationship between cluster belonging and outcomes was also analyzed.

To summarize, the current study aims to:

1. Test how many clusters are needed to describe the sample’s heterogeneity in relation to gender and age stereotype threats;
2. Test if company’s characteristics—such as size (number of employees), percentage of over 50 employees, and percentage of female employees—and employees’ gender and age are associated with the employees’ cluster membership;
3. Test if cluster membership is related to the employees’ work experience, looking specifically at work performance, authenticity, and organizational identification. We expect clusters of workers who score high in gender and/or age stereotype threat to have negative consequences for the considered processes;
4. Test whether age and gender have a moderation effect in the relationship between cluster membership and employees’ outcomes.

## Materials and Methods

### Participants and Procedure

The data for the present study were collected from 33 companies operating in different sectors: Chemical/Pharmaceutical, Insurance, Automotive, Communication, Health Tech, Nuclear Decommissioning, Telecommunications, Utility, Energy/Oil & Gas, Professional Services, Industrial Goods, Fashion and Luxury, Banking, Games, IT/e-Commerce, Postal Services, International Transportation, Food and Beverage, Media and Entertainment, and University and Education. Companies’ size varied from small (between 50 and 250 employees) to very large companies (over 50,000 employees). Companies also varied in their percentage of female and employees over 50. Fourteen companies indicated that women were less than 40% of the total workforce, while only four companies had more than 60% of female employees. Twenty-seven companies indicated that 40% of their workforce included workers over 50, and only one company indicated that more than 60% of its employees were over 50.

A total of 9,195 workers—64% of whom were men—aged between 50 and 69 years old completed the questionnaire (*M* = 55.33, *SD* = 3.29). The participants lived in different areas of Italy (48% in the North, 32% in the Center, and 18% in South and Isles). As for their job title, 5.7% were members of the board of directors, 31.7% managers, 61.8% office clerks, and the rest were skilled or unskilled workers. Women were more likely to be in office clerks jobs than men. Men were more likely to be board members or managers than women [*χ*^2^(3) = 74.47, *p* < 0.001]. The vast majority of the sample was in full-time employment (96%) and permanent positions (97.4%).

As for the length of service, participants declared, on average, to have worked for 31.97 years (*SD* = 4.68) and to have been working for the company in which they were currently employed for an average of 26.93 years (*SD* = 8.06). Participants were contacted by email *via* the Human Resource Department of each company involved in the study. The email presented the study and contained a link to an anonymous online survey.

## Measures

*Age Stereotype Threat*. Perceptions of age stereotype threat in the workplace were assessed by adopting von Hippel et al. (2013) measure of Age-Based Stereotype Threat—previously adapted by measure of stereotype threat of von Hippel et al. (2011a). The five-item scale assesses perceived age-related stereotyping in the workplace, i.e., “People of my age often face biased evaluations in this workplace,” and “I feel that my career options are limited because of my age.” Participants responded using a 5-point scale ranging from 1 (strongly disagree) to 5 (strongly agree). Omega coefficient (ɷ) for this scale was 0.75.

*Gender Stereotype Threat*. Perceptions of gender stereotype threat in the workplace were assessed with an adapted version of the scale developed by Blau and Tatum (2000). We chose this measure in order to have a scale that depicted negative career-related stereotypes, power, and salary structure, as these aspects are known to devalue women in the workplace in Italy (EIGE, 2017). Participants responded using a 5-point scale ranging from 1 (strongly disagree) to 5 (strongly agree). An example of the items presented is “Men/women have greater possibility of career,” “Men/women are given more job responsibilities,” and “Men/women, with the same amount of experience, earn a higher salary for performing the same job.” Omega coefficient (ɷ) for this scale was 0.85 and 0.74, respectively, for women and men.

*Working Performance*. We use a single item to measure the perception of the individual performance—“How do you evaluate your working performance in the last 4 weeks?” Participants were asked to evaluate it on a scale from 0 (worst) to 10 (best) performance.

*Authenticity*. Authenticity was measured by using a six-item self-alienation subscale of the Individual Authenticity Measure Work (van den Bosch and Taris, 2014). Examples of items were “I do not feel who I truly am at work” and “At work I feel alienated.” Responses, given on a 7-point scale ranging from 1 (totally disagree) to 7 (totally agree), were recoded to measure authenticity. The internal consistency of the scale was sufficient (*ɷ* = 0.62).

*Organizational Identification*. Organizational identification was measured utilizing the five items of the Shared Experience subscale of the Mael and Tetrick’s (1992) Organizational Identification scale. Responses were based on a 5-point scale ranging from 1 (totally disagree) to 5 (totally agree). Example items are “I’m very interested in what others think about my organization” and “When I talk about my organization, I usually use ‘We’ rather than ‘They’.” The internal consistency of the scale was *ɷ* = 0.85.

*Company Characteristics*. Company Characteristics were assessed through an *ad hoc* questionnaire, sent to the HR managers, in which they had to indicate the size of the company at the time of data collection (number of individuals employed by the company), the percentage of over 50 employees (on the total number of employees), and the percentage of female employees (on the total number of employees).

## Data Analysis

### Descriptive Statistics

Descriptive statistics of variables involved in the current study were estimated in SPSS (version 20). Normal distribution of item was checked using the criterion set by Muthén and Kaplan (1985), whose items with skewness and kurtosis lower than |1| should be considered normally distributed. The mechanism beyond the missing data was evaluated by performing Little’s MCAR test (Little, 1988); a nonsignificant chi-square indicates that missing data are completely at random (MCAR).

Before running other analyses, we calculated the intraclass correlation coefficient (ICC) to measure the proportion of variance in the outcome variables that is explained by the grouping structure of the data. The ICC values obtained for the working performance (ICC = 0.009), authenticity (ICC = 0.004), and organizational identification (ICC = 0.056) variables indicated that the nested structure of data could be ignored (Dyer et al., 2005).

Items belonging to the same scale were aggregated in a total score performing a Confirmatory Factor Analysis (CFA) and saving the latent factor score of the CFA model, thought Mplus software (version 7). This approach has three advantages over the classic total score obtained averaging items’ scores. First, the final score (latent factor), being free from the measurement error, is a more reliable indicator of the construct. Second, the latent factor score is also estimable for participants who answered just one item of the scale, allowing the researchers to include all participants in the next steps of analysis. Third, the obtained factor score is a standardized indicator, which is useful to identify outliers as well as obtain a more interpretable cluster solution. Following criterion of Tabachnick and Fidell (2013), participants who, on at least one indicator, scored over 3.29 were considered outliers and removed from the dataset.

### Clusters of Gender and Age Stereotype Threats

In order to identify different subgroups (clusters) of people in the sample that shared the same pattern of stereotype threat for gender and age, we performed cluster analysis, adopting the two-step procedure (Gore, 2000) in SPSS software. The observed indicators of this analysis were the two-factor scores, measuring respectively “gender stereotype threat” and “age stereotype threat.”

The first step of the analysis consisted of a hierarchical cluster analysis conducted using Ward’s method of squared Euclidean distance to identify the optimal number of classes. We compared cluster solutions with two, three, four, and five clusters according to three criteria (Milligan and Cooper, 1985): explanatory power—at least 45–50% of the variance of the variables used as indicators of the clusters has to be explained by the cluster solution; parsimony—solutions with less cluster take preference; theoretical meaningfulness of the obtained clusters. In the second step, the initial cluster centers—taken from the cluster solution selected on the basis of the three aforementioned criteria—were used as nonrandom starting points in an iterative k-means clustering method in order to produce a final and more precise cluster solution. Results of this second step consisted of assigning employees to the cluster that better described their patterns of age and gender stereotype threat.

In order to validate the cluster solution, we verified if the different stereotype threat clusters were equally distributed across gender (men vs. women) and age (lower vs. higher than 55 years old) using chi-square tests. Due to the large sample size of this study (*N* = 9,195), a cut-off of *α* = 0.001 was used to define the significance of these and following tests, in order to reduce the possibility of type I error (Little, 2013). In other words, results were considered significant only if their *p* value was lower than 0.001.

### Relationship Between Company’s Characteristics and Employees’ Clusters

In order to verify if the company’s characteristics could be linked to a specific pattern of gender and age stereotype threat, we performed a multinomial logistic regression. The dependent variable was the cluster membership, while the independent variables were the three characteristics of the company expected to affect the likelihood of each stereotype threat cluster frequency. These were identified as the size of the company (number of individuals employed by the company), the percentage of employees over 50 (out of the total number of employees), and the percentage of female employees (out of the total number of employees).

Moreover, we also tested if the relationship between these company’s characteristics and employees’ cluster membership was moderated by the gender of the employees, by performing, separately for male and female employees, the same multinomial logistic regression already tested on the total sample. If the two regression models showed different results, the moderating effect of the gender would be confirmed. The same procedure was replicated for the dichotomous age variable.

### Relationship Between Employees’ Clusters and Outcome Variables

To verify if the stereotype threat cluster to which employees belong is related to their outcomes, we performed one analysis of variance (ANOVA) for each employees’ outcome. In particular, in the ANOVA, the measure of employees’ outcome (working performance, authenticity, or organizational identification) was included as a dependent variable, while three independent variables were included: cluster membership, gender, and age. To test if the stereotype threat cluster membership affected the employees’ outcomes, the significance of the cluster main effect was checked, and, when needed, *post hoc* analyses (Tukey method) measured the extent of which the relationship between any two clusters variables differed. To test the moderator effect of gender and age on the relationship between clusters and outcome variables, the interaction effects were evaluated.

## Results

### Descriptive Statistics

In Table 1, descriptive statistics of the items measuring gender and age stereotype threat are reported. Each item has a response rate that ranges from 98.87% (item 2 of the gender stereotype threat scale) to 99.78% (item 1 of the gender stereotype threat scale), and the missing data were not completely at random: *χ*^2^(225) = 329.15; *p* < 0.001. In the CFA models, applied to estimate the factor score of each construct of interest (gender stereotype threat and age stereotype threat), the missing data were processed using the full information maximum likelihood (FIML) method.

**Table 1:** Descriptive statistics of items and latent factors measuring gender and age-based stereotype threats.

		*N*	Min	Max	*M*	*SD*	Skewness	Kurtosis
Gender stereotype threat	Item 1	9,175	1	4	2.32	0.89	0.41	−0.54
	Item 2	9,091	1	4	2.17	0.92	0.61	−0.36
	Item 3	9,125	1	4	1.97	0.89	0.71	−0.20
	Latent factor	9,195	−1.18	3.19	−0.001	0.93	0.99	0.32
Age stereotype threat	Item 1	9,151	1	5	1.84	1.03	0.97	−0.01
	Item 2	9,139	1	5	1.75	1.02	1.26	0.71
	Item 3	9,155	1	5	3.51	1.29	−0.66	−0.64
	Item 4	9,138	1	5	2.94	1.25	−0.04	−0.97
	Item 5	9,132	1	5	2.02	1.14	0.86	−0.25
	Latent factor	9,195	−1.35	2.10	−0.001	0.94	0.63	−0.22

As reported in Table 1, minimum and maximum scores on both latent factors did not exceed 3.29, and consequently, no outliers were identified. The total sample on which further analyses were performed included 9,195 cases.

### Clusters of Gender and Age Stereotype Threat

As reported in Table 2, results of the cluster analysis showed that the three-cluster solution was the first explanatory power criterion, explaining at least 45–50% of the variance of gender and age stereotype threat scores.

**Table 2:** Percentage of indicators showing variance for each cluster analysis solution (*N* = 9,195).

	2-class	3-class	4-class	5-class
Gender stereotype threat	0.65	0.66	0.66	0.76
Age stereotype threat	0.01	0.49	0.69	0.69

The same criterion was satisfied also by four‐ and five-class solutions, but for the parsimony criterion, the three-class solution was preferred. The last criterion to evaluate was the theoretical meaningfulness of the clusters. Based on this criterion, we selected the four-class solution. Specifically, for the three-cluster solution, the interpretation of the clusters identified was the following: the first cluster grouped employees threatened by gender only, the second cluster grouped employees threatened by age only, while the third cluster grouped employees who did not perceive any kind of stereotype threat. From a theoretical point of view, this configuration misses the patterns in which the employees reporting stereotype threat for both gender and age. Consequently, we selected the four-cluster solution as the best model, and we ran the second step of the cluster analysis using this approach. The final four-cluster solution allowed us to explain the 70 and 72% of gender stereotype threat and age stereotype threat variance, respectively. The four obtained clusters were theoretically meaningful (see Supplementary Figure 1).

The first cluster grouped 758 employees (8.2%) who perceived both gender and age stereotype threat. The second cluster included 1,735 employees (18.9%) who perceived only gender stereotype threat, while the third cluster (*N* = 2,013; 21.9%) grouped those employees who perceived only age stereotype threat. The cluster with the larger size was the fourth, as half of the participants (*N* = 4,689; 51.0%) did not perceive any kind of stereotype threat.

To validate this cluster solution, we verified if gender and age were randomly distributed across the four obtained clusters. As expected, the gender was nonrandomly distributed across clusters [*χ*^2^(3) = 4,089.79; *p* < 0.001; Cramer’s *V* = 0.67] as women were more likely to report higher levels of threat for either gender alone or both gender and age stereotype. Men were more likely to feel threatened exclusively by age-related stereotypes or not feel threatened at all. Age was also closely related to the cluster membership. In particular, by recoding the age variable in a dichotomous variable using the mean and median value (55 years old), we found that the age class is significantly related to cluster membership [*χ*^2^(3) = 4,089.79; *p* < 0.001; Cramer’s *V* = 0.67].

People aged 55 or younger (*N* = 4,344, 54.9%) were less likely to belong to the cluster whose perceived threat was related to age and more likely to belong to the cluster who saw the threat as related to gender. Conversely, people older than 55 (*N* = 35.70, 45.1%) were less likely to be within the cluster whose threat was gender and more likely to be part of the cluster whose perceived threat was age.

### Relationship Between Company’s Characteristics and Employees’ Clusters

In this section, we determined whether the company’s characteristics—size, percentage employees over 50, and percentage of female employees—were related to the employee clusters identified and if this relationship is moderated by employees’ gender and age. The multinomial logistic regression applied to test that association with the size of the company—percentage of employees over 50 and percentage of female employees on the employees’ cluster membership—indicated that the company’s characteristics significantly affected how employees perceived stereotype threat for gender and age: likelihood ratio test:*χ*^2^(9) = 139.79; *p* < 0.001; Nagelkerke pseudo *R*^2^ = 0.018. The only predictor that was significant per *p* < 0.001 was the percentage of employees over 50 (see Table 3). Specifically, having a higher number of over 50s (OR = 0.98) reduced the likelihood of employees belonging to the cluster of those high in both gender and age stereotype threat and increased the likelihood of being within the cluster in which no stereotype threat was perceived (reference group). In other words, in companies where the percentage of over 50s was higher, employees perceiving simultaneously high gender and age stereotype threat was less likely.

**Table 3:** Summary of multinomial logistic regression analysis for the company’s characteristics—prediction of membership of stereotype threat cluster (*N* = 9,195).

Group^a^	Variable	*Β*	*SD*	Wald^b^	*p*	OR	95% CI
Both	Constant	−0.97	0.30	10.45	0.001		
	Company size	0.00	0.00	6.57	0.01	1.000	[1.00 1.00]
	% of over 50	−0.02	0.01	14.10	<0.001	0.98	[0.97 0.99]
	% of female	−0.01	0.01	0.15	0.70	1.00	[0.99 1.01]
Gender only	Constant	−0.86	0.23	13.90	<0.001		
	Company size	0.00	0.00	3.33	0.07	1.00	[1.00 1.00]
	% of over 50	−0.01	0.01	3.70	0.05	0.99	[0.99 1.00]
	% of female	0.01	0.01	2.14	0.14	1.01	[1.00 1.01]
Age only	Constant	0.12	0.21	0.34	0.56		
	Company size	0.00	0.00	6.09	0.01	1.00	[1.00 1.00]
	% of over 50	−0.01	0.01	10.51	0.001	0.99	[0.98 1.00]
	% of female	−0.01	0.01	11.39	0.001	0.99	[0.98 0.99]

OR, odd ratio; CI, confidence interval.

a The reference group is None.

b *df* = 1.

To test if the relationship between a company’s characteristics and employees’ cluster membership was moderated by gender, we rerun the same multinomial logistic regression separately for men and women. Although the company’s characteristics proved to have a significant impact on both men’s [likelihood ratio test: *χ*^2^(9) = 77.84; *p* < 0.001] and women’s [likelihood ratio test:*χ*^2^(9) = 146.54; *p* < 0.001] stereotype threat clusters, their impact was stronger for women (Nagelkerke pseudo *R*^2^ = 0.056) than for men (Nagelkerke pseudo *R*^2^ = 0.017). While for men, no independent variable, taken individually, was sufficient to determinate a change in the cluster membership, for women, the percentage of female employees significantly affected (*p* < 0.001) cluster membership (see Table 4).

**Table 4:** Summary of multinomial logistic regression analysis for the company’s characteristics-prediction of membership of stereotype threat cluster within the women subsample (*N* = 3,271).

Group^a^	Variable	*Β*	*SD*	Wald^b^	*p*	OR	95% CI
Both	Constant	2.14	0.44	23.27	<0.001		
	Company size	0.00	0.00	4.03	0.045	1.00	[1.00 1.00]
	% of over 50	−0.01	0.01	3.80	0.051	0.99	[0.97 1.00]
	% of female	−0.04	0.01	37.52	<0.001	0.96	[0.94 0.97]
Gender only	Constant	2.34	0.38	37.58	<0.001		
	Company size	0.00	0.00	0.44	0.51	1.00	[1.00 1.00]
	% of over 50	−0.01	0.01	0.35	0.55	1.00	[0.98 1.01]
	% of female	−0.04	0.01	41.72	<0.001	0.96	[0.95 0.97]
Age only	Constant	−0.53	0.57	0.88	0.35		
	Company size	0.00	0.00	6.16	0.01	1.00	[1.00 1.00]
	% of over 50	0.01	0.01	0.01	0.94	1.00	[0.98 1.02]
	% of female	−0.01	0.01	0.82	0.36	0.99	[0.98 1.01]

OR, odd ratio; CI, confidence interval.

a The reference group is None.

b *df* = 1.

In particular, a higher number of female employees reduced the likelihood of women featuring in the cluster high in gender stereotype threat (OR = 0.96) or both gender and age stereotype threat (OR = 0.96) and increase the likelihood of being part of clusters that perceived no threats.

Finally, to verify if the employees’ age moderated the relationship between a company’s characteristics and cluster membership, we rerun the same multinomial logistic regression separately for employees aged over or under 55. Although the company’s characteristics showed a significant impact on both employees aged 55 or younger [likelihood ratio test:*χ*^2^(9) = 65.39; *p* < 0.001] and over 55 employees [likelihood ratio test:*χ*^2^(9) = 77.35; *p* < 0.001], their impact proved to be slightly more significant for older (Nagelkerke pseudo *R*^2^ = 0.026) than younger employees (Nagelkerke pseudo *R*^2^ = 0.018).

At the same time, in both subgroups, no independent variable, taken individually, was sufficient to determinate a significant (*p* < 0.001) change in the cluster membership. As for the total sample, having a higher number of employees over 50 reduced (OR = 0.98; *p* < 0.001) the likelihood of being in the cluster high on stereotype threat for both gender and age compared to the cluster where no stereotype threat was perceived. This relationship was checked in the two age subgroups.

In the subgroup of employees aged 55 or less, this relation was nonsignificant for *α* = 0.05 as Wald test (1) = 1.81; *p* = 0.18, while in the subgroup of employees over 55, this relation was significant for *α* = 0.01, as Wald test (1) = 8.94; *p* = 0.003. Therefore, it can be stated that the significant (*p* < 0.001) relationship found within the total sample was mainly ascribable to the older employees.

### Relationship Between Employees’ Clusters and Outcome Variables

In this section, we established the relationship between employees’ clusters and their work outcomes—working performance, authenticity, and organizational identification—and if this relationship was affected by gender and/or age. We focused exclusively on aspects within the scope of the aim of the study—the main effect of the cluster variable (impact of cluster membership on employees’ outcome), the interaction effect of cluster*gender (moderating effect of gender), and the interaction effect of cluster*age (moderating effect of age). Further detail about the other effects—all nonsignificant per *α* = 0.001—is available on request.

First, we tested the relationship between employees’ cluster membership and their performance. Results suggested that the kind of stereotype threat pattern that the employees perceived affected the employees’ working performance: *F*(3, 7,861) = 52.40; *p* < 0.001; *η*^2^ = 0.020. In particular, *post hoc* analysis revealed that the highest levels of performance (see Table 5) was reported by employees who were not subject to any kind of stereotype threat or who only perceived stereotype threat related to gender. Their levels of performance were significantly higher than the performance level reported by those who were high in both gender and age stereotype threat. Finally, those with the lowest level of performance were those employees whose stereotype threat was age related. This relationship was neither moderated by gender [interaction effect cluster*gender: *F*(3, 7,861) = 2.00; *p* = 0.11] nor age [interaction effect cluster*age: *F*(3, 7,861) = 0.56; *p* = 0.64].

**Table 5:** Mean of dependent variable separately for each cluster.

Cluster	Working performance	Authenticity	Organizational identification
Both	7.57 (*SD* = 1.34; *n* = 652)	5.44 (*SD* = 1.11; *n* = 643)	3.36 (*SD* = 0.96; *n* = 653)
Gender only	8.01 (*SD* = 1.11; *n* = 1,504)	5.87 (*SD* = 0.98; *n* = 1,489)	3.63 (*SD* = 0.88; *n* = 1,506)
Age only	7.39 (*SD* = 1.37; *n* = 1,713)	5.41 (*SD* = 1.04; *n* = 1,689)	3.41 (*SD* = 0.89; *n* = 1,716)
None	7.99 (*SD* = 1.13; *n* = 4,008)	5.90 (*SD* = 0.87; *n* = 4,006)	3.79 (*SD* = 0.82; *n* = 4,023)
*Total sample*	7.83 (*SD* = 1.23; *n* = 7,877)	5.75 (*SD* = 0.98; *n* = 7,827)	3.64 (*SD* = 0.88; *n* = 7,898)

SD = standard deviation; *n* = sample size.

Regarding authenticity, the employee’s stereotype threat pattern explained the 3.3% of the employee’s authenticity: *F*(3, 7,811) = 90.00; *p* < 0.001; *η*^2^ = 0.033. Specifically (see Table 5), the levels of authenticity reported by employees who did not perceive any kind of stereotype threat or gender stereotype threat alone were significantly higher than the levels of authenticity reported by employees who viewed both age and gender as a stereotype threat.

Furthermore, gender significantly moderated the relationship between the cluster membership and employees’ authenticity [interaction effect cluster*gender: *F*(3, 7,811) = 14.79; *p* < 0.001; *η*^2^ = 0.006]. In particular (see Supplementary Figure 2), the four stereotype threat clusters and their level of authenticity varied quite significantly among women and men. Within the men subsample, the highest levels of authenticity were reported when no stereotype threat was perceived (*M* = 5.89; *SD* = 0.87). Lower authenticity levels were reported when men felt gender stereotype threat (*M* = 5.44; *SD* = 1.12) or age stereotype threat (*M* = 5.37; *SD* = 1.03), while the lowest levels were reported when men felt both gender and age stereotype threat (*M* = 4.77; *SD* = 1.14). On the other hand, within the women subsample, the gender stereotype threat did not seem to affect women authenticity. Women who perceived gender stereotype threats (*M* = 5.92; *SD* = 0.95) had the same level of authenticity as women who did not feel any stereotype threat at all (*M* = 5.97; *SD* = 0.89). Instead, women perceiving age stereotype threat only (*M* = 5.58; *SD* = 1.03) or both for age and gender (*M* = 5.54; *SD* = 1.07) had significantly lower level of authenticity. To sum up, while for men, gender and age stereotype threat have the same negative impact on authenticity, for women, only age stereotype threat harms authenticity. The employees’ age did not moderate the relationship between cluster membership and authenticity [interaction effect cluster*age: *F*(3, 7811) = 1.29; *p* = 0.28].

Finally, we tested the relationship between the cluster membership and the employee’s organizational identification and established a significant relationship: *F*(3, 7882) = 53.68; *p* < 0.001; *η*^2^ = 0.020. In particular (see Table 5), the highest levels of identification with the company were reported by employees who did not perceive any kind of stereotype threat. Employees who scored high in gender stereotype threat alone showed the second-highest level of identification with the company. Those employees who scored high in either age-only stereotype threat or both age and gender reported the lowest level of organizational identification. This relationship was neither moderated by the employees’ gender [interaction effect cluster*gender: *F*(3, 7882) = 1.06; *p* = 0.37] nor age [interaction effect cluster*age: F(3, 7882) = 1.17; *p* = 0.32].

## Discussion

The present study on the effects of age and gender stereotype threat on worker’s identity processes and performance expands the current understanding of this important topic by examining the combined effect of these two forms of negative stereotypes using a person-centered approach.

The cluster analysis in a large sample helps identify groups of workers based on their perception of the stereotype threat. It is important to highlight these data in organizations because they demonstrate the perceived vulnerability of the population over 50 in their work context. It is also interesting to note that most workers felt threatened by negative stereotypes related to age (30.1%) compared to those related to gender. Therefore, ageism is a truly significant issue at work and needs to be tackled effectively. In this context, women are in a particularly difficult situation, especially those in organizations in which they are underrepresented, as they are more likely to be subject to double stereotype threats—gender as well as age.

The presence of high age stereotype threat levels is demonstrated to be linked to the worse performance outcomes. People who are doubly threatened—age and gender—or who experience age stereotype threat alone claimed the lowest levels of identification with the organization and authenticity scale. In the workplace, feeling marginalized because of age discrimination can trigger a negative process that is likely to lead workers to perform poorly, identify less closely with the organization, and achieve a lesser sense of authenticity.

The findings of this study are in line with the plentiful literature available on the subject that demonstrates the negative impact of age-based stereotype threat on workers over 50 (e.g., von Hippel et al., 2013). Gender stereotype threat seems to have a less significant impact on performance and identity. Hence, workers perceiving high levels of gender stereotype threat alone did not report lower levels of performance and authenticity compared to those who do not feel threatened at all. There is only a difference in the level of organizational identification, in line with previous findings on analyzing gender linguistic bias and job identification in women (Stout and Dasgupta, 2011). A possible explanation is that the negative stereotypes related to age are more harmful in work contexts than those related to gender—that is certainly true for workers over 50. According to the *stereotype content model* (Fiske et al., 2002), older adults can be classified as low in skill, making them a low-performance group. Thus, being branded “less efficient” due to age may be more debilitating for the older worker than any “male/female” bias. In the case of age stereotype threat, older workers are more likely to self-fulfill this prophecy, becoming less effective in their work (McCann and Giles, 2002).

Another possible explanation of this particular finding may derive from a lack of acceptance of one’s own aging, exposing the individual to this type of threat. Previous studies have shown that higher levels of identification with the threatened identity group—in our case age identity—lessens the negative effects of stereotype on the individual’s outcomes (Bergeron et al., 2006).

All considered, changing the social and organizational view of age might minimize the burden of belonging to a stigmatized group, and it might lead to an improvement in the identity processes of older workers. Furthermore, fighting ageism would directly benefit organizations in their mission to redress job performance among older workers. The practical benefit of such results may contribute to a positive change in the image of a cluster of workers. If groups are viewed less stereotypically, companies would increase the sense of belonging and organizational engagement (Filstad et al., 2019), the levels of collaborations among workers, and the sharing of experiences and knowledge throughout the organization—all key elements of mutual benefit and development.

An intriguing finding of this study is the significant difference between men and women’s authenticity while facing age stereotype threat. Indeed, it seems that men are much more affected in their authenticity in the workplace while facing stereotype threat. This result contrasts with the previous findings that showed that, in order to avoid devaluation in the workplace, middle-aged women identified themselves more readily with younger generations compared to men (Barrett, 2005). A possible interpretation of this evidence is that women in work contexts face gender stereotype more frequently and throughout their professional life, developing more effective coping strategies as part as their identity process.

Further studies should analyze this finding in more depth. Nevertheless, it is important to note that ageism in the workplace seems to be more harmful to men’s authenticity than for women’s. Coaching has been an effective tool in achieving higher levels of authenticity among women in the workplace (Jackson, 2019); hence, such approach may also be used to aid older male workers.

The limitations of this study must also be underlined. First, the use of a correlational and cross-sectional design prevented the establishment of causality. Future research should examine the causal nature of these relationships with longitudinal designs. Furthermore, the measure used to assess stereotype threat could be improved and refined to explore this dimension further. Future studies should replicate these findings using different measures and methodologies. Nevertheless, our study has high ecological validity, in that it analyzes the effects of age and gender stereotype threat outside of formal test-based settings, giving important evidence on how much stereotype threat may impact negatively on an individual’s work life. Previous studies demonstrating the relationship between poor performance and stereotype threat have taken place exclusively in laboratory settings. Very little research has examined the antecedents and consequences of prolonged stereotype threats outside the laboratory, and almost no research has ever been carried out in organizational settings (Kalokerinos et al., 2014). This study is one of the few pieces of research that focused on stereotype threats in the workplace and analyzed their effects on a large sample of workers. Moreover, this study provides a first insight into how gender and age stereotype threat may affect workers over 50 in Italy. Finally, we must underline that our exploratory study is related to the context of inquiry that we have chosen. Cultural differences play a strong role in determining perception. Organizations and groups are immersed in cultures and subcultures that influence their perceptions, expectations, and individual evaluations about personal and work experience. In further studies, it would be useful to explore the cultural aspects and their practical applications to individual and group routines and habits. These have the power to affect stereotype threats in a virtuous or vicious circle (see for example Manzi et al., 2020). That said, we intended to provide a blueprint for similar studies, as it could be adapted according to contextual and situational differences. Although if focused on “the cultural side,” this study can represent a means of cultural change.

In conclusion, the analysis presented above aims to aid theoretical advancement and its practical implications in the organizational and management field. From a theoretical point of view, it is important to study the effects of two forms of negative stereotypes to highlight and understand their combined risk. Regarding practical implications, as a result of the evidence gathered in the study, new HR management orientations and policies may be introduced to lessen the power of stereotype threats. Consequently, new organizational policies to support men and women in their strategies to cope with stereotypes should be developed.

On the one hand, it would be beneficial to undertake an ongoing internal dialogue and an open organizational debate around stereotypes by, for example, introducing coaching and training programs for engendering an inclusive culture in the workplace. On the other, new organizational practices could directly curb the effect of stereotype threat on workers’ engagement. Typically, diversity-conscious practices are particularly beneficial in counteracting negative attitudes that young managers had toward older colleagues, whereas diversity blind practices were especially helpful in generating engagement among older workers with older managers (Kulik et al., 2016). The key point to consider in tackling age diversity is to focus organizational practices on the performance and skills rather than age, with a view to shifting the attention away from stereotype itself.

## Data Availability Statement

The raw data supporting the conclusions of this article will be made available by the authors, without undue reservation.

## Ethics Statement

Ethical review and approval was not required for the study on human participants in accordance with the local legislation and institutional requirements. The participants provided their written informed consent to participate in this study.

## Author Contributions

CM, ST, and MG conceived the study and collected the data. CM, ER, and MG wrote the first draft of the manuscript. AS run the analysis. All authors discussed the results and contributed to the final manuscript.

### Conflict of Interest

The authors declare that the research was conducted with no commercial or financial relationships that could be construed as a potential conflict of interest.

## Supplementary Material

Supplementary Figure 1 Mean latent factor scores of gender and age stereotype threat for the 4-cluster solution.

Supplementary Figure 2 Mean levels of authenticity separately for discrimination cluster (both, only gender, only age, none) and gender (men, women).
