# Supplementary material for: Double Jeopardy-Analyzing the Combined Effect of Age and Gender Stereotype Threat on Older Workers
Source: Front Psychol. 2021 Jan 12;11:606690. doi: 10.3389/fpsyg.2020.606690 (PMC7835537; doi:10.3389/fpsyg.2020.606690)
Supplement: Supplementary Figure 1 — Mean latent factor scores of gender and age stereotype threat for the 4-cluster solution. [file Data_Sheet_1.docx]

Supplementary Material


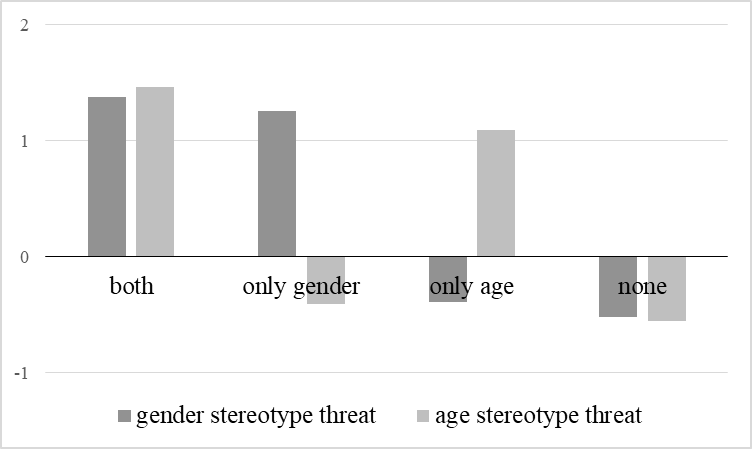


*Figure 1.* Mean latent factor scores of gender and age stereotype threat for the 4-cluster solution


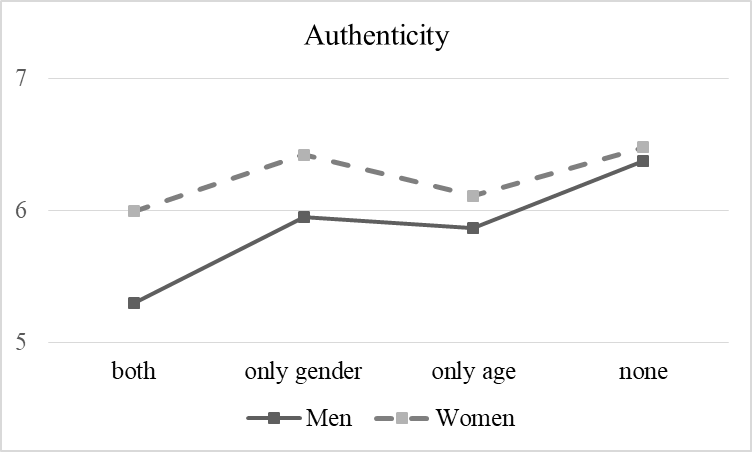


*Figure 2.* Mean levels of authenticity separately for discrimination cluster (both, only gender, only age, none) and gender (men, women).
